# Morphine and kisspeptin influences on 5-α reductase and aromatase gene expression in adult male rats

**DOI:** 10.22038/IJBMS.2019.14053

**Published:** 2019-12

**Authors:** Homayoun Khazali, Fariba Mahmoudi

**Affiliations:** 1 Department of Animal Science and Biotechnology, Faculty of Life Sciences and Biotechnology, Shahid Beheshti University, Tehran, Iran; 2 Faculty of Sciences, University of Mohaghegh Ardabili, Ardabil, Iran

**Keywords:** Aromatase, Hypothalamus, Kisspeptin, Liver, Morphine, Testis, 5α-reductase

## Abstract

**Objective(s):**

Kisspeptin and opioids are important factors for controlling GnRH/LH secretion. In present study, influences of kisspeptin or morphine were investigated on 5α- reductase or aromatase (*CYP19*) genes expression in the hypothalamus, testis and liver. It aimed to investigate whether kisspeptin pathway may control morphine effects on plasma concentration of testosterone.

**Materials and Methods:**

Twenty adult male rats in four groups received saline/saline, kisspeptin (1 nmol)/saline, morphine (5 mg/kg)/saline or kisspeptin/morphine respectively. Mean relative 5α-reductase and aromatase mRNA levels were determined by RT-PCR.

**Results:**

Morphine/saline injection increased significantly mean relative mRNA levels of hypothalamic 5α-reductase or aromatase compared to saline/saline. While morphine/saline did not alter mRNA levels of them compared to saline/saline group in the testis and liver. Kisspeptin/saline did not significantly decrease mean relative 5α-reductase or aromatase genes expression compared to saline/saline group in the hypothalamus, testis and liver. Injections of kisspeptin/morphine did not significantly decrease mean relative 5α-reductase or *aromatase *genes expression compared to morphine/saline group.

**Conclusion:**

Up-regulation of hypothalamic 5α-reductase or aromatase mRNA levels may partly induce the inhibitory effects of morphine on GnRH/LH release. Different effects of morphine on aromatase or 5α- reductase genes expression levels in the liver and testis compared to brain may be partly due to different sensitivity or functions of them to morphine used dose.

## Introduction

Hypothalamus - pituitary- gonadal (HPG) axis is under precise control of steroid hormones synthesized by gonads or peripheral organs. In addition to testosterone, 17β estradiol or dihydrotestosterone (DHT) play important role in regulation of gonadotropin releasing hormone (GnRH) and luteinizing hormone (LH) secretion in males or females (1, 2). Testosterone produced intra testes could be converted to different steroids following entrance to peripheral organs like liver, brain, prostate, skin, and adipose tissue (3). Aromatase is an enzyme which is encoded by *Cyp19* gene and it converts testosterone to 17β estradiol in the brain, testis, ovary, liver, breast and so on (3). The estradiol produced by the action of aromatase is completely necessary for normal development of testis, spermatogenesis, brain sexual differentiation, exerting negative feedback mechanism control on tonic release of GnRH/LH and following plasma testosterone concentration in males (3).

The 5α- reductase enzyme is found mainly in two isozymes including 5α- reductase type 1(5αRI) and 2(5αRII) composing of 259 and 254 amino acids in several organs respectively (4). Both isozymes convert testosterone to DHT by reducing the double bound between carbon 4 and 5 of testosterone. However DHT produced by the isozymes of 5α- reductase exert different effects on tissues functions expressing them. In fact, 5α- reductase II is mainly synthesized in sexual organs and it is associated with embryonic sexual differentiation of brain and external genitals. While 5α- reductase I is extremely expressed throughout the body and it is mainly related to steroid catabolization especially in the liver and central nervous system (4). That is to say, testosterone is not the only substrate for 5α- reductase enzymes because they could reduce progesterone, aldosterone, cortisol, androstendione and so on (2, 3). The DHT effects on HPG axis preferentially exert at hypothalamic levels and its injections decreases LH and FSH secretion following inhibiting hypothalamic GnRH release in normal individuals (5). On the other hand DHT produced intra hypothalamus partly participates in tonic release pattern of GnRH/LH hormones (5).

Morphine is an extracted alkaloid from poppy plant and as a potential mu (µ) type receptor agonist. It mimics the most physiological functions of endogenous β-endorphin. It induces infertility dominantly via decreasing the GnRH neurons activity rather than direct influences on gonadotrophs or leydig cells (6). Most of the previous researches have been shown that morphine or other opioids decline plasma testosterone levels predominantly via suppressing hypothalamic GnRH/LH release. (6). However, a few researches demonstrated that morphine treatment may decline the plasma testosterone level by increasing its metabolism peripherally (7, 8). 

Kisspeptin and its G protein-coupled receptor, GPR54, are necessary for normal pubertal development and HPG axis function (9, 10). Disorder or mutations in hypothalamic kisspeotin or GPR54 receptor signaling pathway disrupt GnRH/LH secretion and results in pubertal delay or infertility in humans and other species (11). In the brain, kisspeptin is found in four different types including kisspeptin54, 14, 13 and 10 amino acids which they have similar affinity to GPR54 receptor. Based on several studies, central or peripheral injections of kisspeptin stimulate the secretion of HPG axis hormones (11, 12). Stimulatory effects of kisspeptin on GnRH/LH completely suppressed by the injection of GPR54 receptor antagonist named peptide 234 (11).

Mu opioid receptor are not expressed on GnRH neurons of arcuate nucleus (ARC) of hypothalamus which is responsible for controlling the tonic release of GnRH/LH and following testosterone synthesis (13). Most of previous studies established that the inhibitory influences of β-endorphin or morphine on the tonic release of GnRH/LH and subsequently gondal steroid hormones are exerted via indirect intra hypothalamic neurons including noradrenergic, dopaminergic, GABAergic or kisspeptin neurons (12). Also, central or peripheral information to the reproductive axis are partly relayed by kisspeptin (14). In the present study the effects of kisspeptin or morphine were investigated on 5α- reductase and aromatase genes expression in hypothalamus, testis or liver and it aimed to investigate whether the kisspeptin pathway may control morphine effects on plasma concentration of testosterone.

## Materials and Methods

***Animals***

In the present experimental study, 20 male Wistar rats weighing 230- 250 g (provided by the Center of Neuroscience Research of Shahid Beheshti University, Iran) were housed in the cages under controlled temperature (22± 2 C°) and light (12 hr light/ dark cycle, light on at 0700). Animals had free access to food and water all the time. All procedures for the maintenance and the use of experimental animals were executed with the Guide for the Care and Use of Laboratory Animals (National Institute of Health Publication No. 80-23, revised 1996) and were approved by the Ethical committee of Neuroscience Research Center of Shahid Beheshti University of Medical Sciences (Tehran, Iran).

***ICV cannulation and injections***

Intraperitoneal (IP) injections of a mixture of ketamine and xylezine (ketamine 80 mg/kg BW+ xylezine 10 mg/ kg BW) were used to anesthetize rats. A 22- gauge stainless cannulae was implanted in the third cerebral ventricle (AP=-2.3, ML=0.0, DV=6.5)(12). In 7^th^ day following implantation the cannulae, the drugs including saline (3 µl, ICV)+saline (200 µl, SC), kisspeptin (1 nmol/3 µl, ICV)+saline (200 µl, SC), saline (3 µl, ICV)+morphine (5 mg/kg, 200 µl, SC) or kisspeotin (1 nmol/3 µl, ICV)+morphine (5 mg/kg, 200 µl, sc) were injected into rats (n=5 in each group) respectively. Kisspeptin10 (Ana Spec Co, U.S.A.) and morphine sulfate (Temad Co, Iran) were dissolved in distilled water and they were injected at 09:00- 9:30. 

***Dissections and real-time polymerase chain reaction (RT-PCR)***

Animals were decapitated and hypothalamus, testis and liver samples were dissected and stored at -80 °C. Total RNA was isolated by using the acid guanidinium thiocyanate-phenol-chloroform extraction method (PureZol, Bio RAD, U.S.A). Changes in mRNA levels were investigated by using the Corbette Rotor Gene 6000 apparatus, Korea and SYBR Green I kit in a final volume of 25 µl (Takara Bio Inc., Japan). Reverse transcriptase step used temperatures were as following: 65 °C for 5 min, 42 °C for 60 min and 85 for 5 min (Vivantis Co, Malasia). The PCR cycling conditions were as following: first denaturation 95 °C for 3 min, followed by 40 cycles of denaturation at 95 °C for 30 sec, annealing at 60 °C (5α- reductase or aromatase) and 58 °C (*GAPDH*) for 30 sec and extension at 72 °C for 30 sec, followed by final extension 72 °C for 7min. Forward and reverse sequences of primers used were: *5α- reductase* forward:5′-CGTCCTGCTGGCTATGTTTC-3′ and reverse: 5′- GAAGGCCAAGACAAAGGTGA -3′(7) *aromatase* forward:5′-CGTCATGTTGCTTCTCATCG-3′ and reverse: 5′-TACCGCAGGCT

CTCGTTAAT-3′(15) and *GAPDH* forward: 5′AAGAAGGTGGTGAAGCAGGCATC -3′ and forward: 5′-CGAAGGTGGAAGAGTGGGAGTTG-3′ (12). The 5α-reductase, aromatase and GAPDH ampliﬁed products were 108, 149 and 112 base pairs respectively. Calculation of relative expression levels of the target mRNAs were calculated by the equation 2^-ΔΔCT^.

***Statistical analysis***

The SPSS software was used to evaluate the results by using one- way ANOVA followed by *post hoc* Tukey test. The results are shown as mean±SEM. Significance was reported by *P*≤0.05 

## Results

***Influence of morphine and kisspeptin on hypothalamic mRNA levels of aromatase***

**Figure 1:**
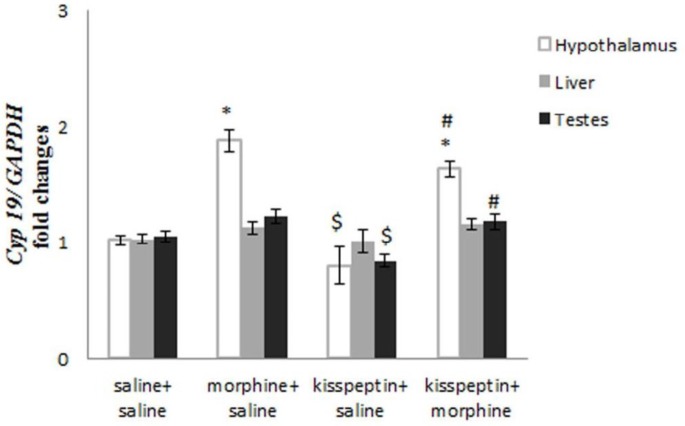
Influences of morphine, kisspeptin or administration of kisspeptin plus morphine on mRNA levels of aromatase (*CYP19*) in the hypothalamus, liver and testis *: compared to saline+saline; $: compared to morphine+saline; #: compared to kisspeptin+saline

Mean mRNA levels of aromatase significantly augment following the infusion of morphine+saline compared to saline+saline group (*P*≤0.05, Figure 1). Kisspeptin +saline injection did not significantly suppress mean mRNA levels of aromatase in comparison to saline+ saline receiving rats (Figure 1). In kisspeptin+morphine receiving rats a significant increase was shown in the mean mRNA levels of aromatase in comparison to saline+saline group (*P≤*0.05, Figure 1). Injection of kisspeptin+saline exerts a significant inhibitory effect on mRNA levels of aromatase compared to morphine+ saline group *(P*≤0.05, Figure 1). Simultaneous infusion of kisspeptin+morphine did not significantly suppress mRNA levels of aromatase compared to morphine+ saline receiving rats (Figure 1). Injections of kisspeptin +morphine induced a significant augment in mRNA levels of aromatase in comparison to kisspeptin+saline (*P*≤0.05, Figure 1).

**I** ***nfluence of morphine and kisspeptin on mRNA levels of aromatase in liver***

Morphine+saline or kisspeptin+morphine did not induce a statistically significant increase in mean mRNA levels of aromatase in comparison to rats receiving saline+saline (Figure 1). Infusion of kisspeptin+saline did not suppress mean mRNA levels of aromatase in comparison to rats receiving saline+saline (Figure 1). Mean mRNA levels of aromatase did not decreased following kisspeptin+saline or kisspeptin+morphine injections in comparison to rats receiving morphine+ saline (Figure 1). Kisspeptin+morphine did not induce a statistically augment in mean mRNA levels of aromatase in comparison to kisspeptin+saline (Figure 1).

***Influences of morphine and kisspeptin on mRNA levels of aromatase in testis***

Mean mRNA levels of aromatase in rats receiving morphine+saline or kisspeptin+morphine did not increase comparing to saline+saline (Figure 1). Kisspeptin+saline did not statistically suppress mean mRNA levels of aromatase comparing to saline+saline group (Figure 1). Mean mRNA levels of aromatase in rats receiving kisspeptin+saline or kisspeptin+morphine declined comparing to morphine+saline but decrease level only in kisspeptin+saline group was statistically significant (*P*≤0.05, Figure 1). Kisspeptin+morphine induced a significant increase the mean relative in mean mRNA levels of aromatase comparing to kisspeptin+ saline (Figure 1).

***Influence of morphine and kisspeptin on hypothalamic 5α-reductase mRNA levels***

**Figure 2:**
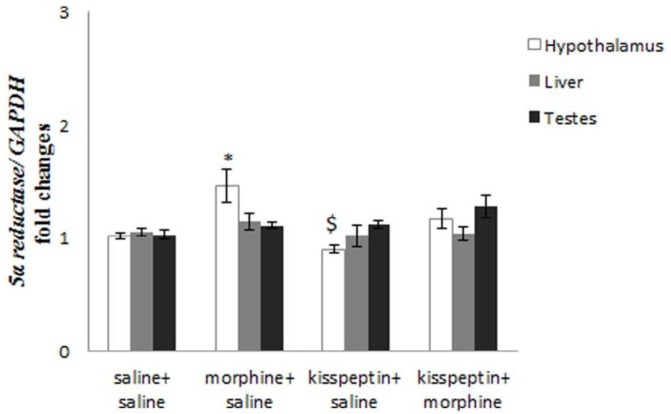
Influences of morphine, kisspeptin or administration of kisspeptin plus morphine on mRNA levels of 5α-reductase in the hypothalamus, liver and testis. *: compared to Saline+saline; $: compared to morphine+saline; #: compared to kisspeptin+saline

Morphine+saline increased significantly mean mRNA levels of 5α-reductase comparing to saline+saline group (*P*≤0.05, Figure 2). Kisspeptin+saline did not decline mean mRNA levels of 5α-reductase in comparison to saline+saline group (Figure 2). Injections of kisspeptin +morphine did not increase the mean mRNA levels of 5α-reductase in comparison to saline+saline group (Figure 2). The mean mRNA levels of 5α-reductase in rats receiving kisspeptin+saline significantly decreased comparing to morphine+saline (*P*≤0.05, Figure 2). The mean mRNA levels of 5α-reductase in rats receiving kisspeptin+morphine did not decrease comparing to morphine+saline (Figure 2). Injections of kisspeptin+ morphine did not increase the mean mRNA levels of 5α-reductase compared to kisspeptin+saline (Figure 2). 

***Influence of morphine and kisspeptin on 5α-reductase mRNA levels in the liver***

The mean mRNA levels of 5α-reductase in rats receiving morphine+saline, kisspeptin+saline or kisspeptin+morphine did not increase comparing to saline+saline (Figure 2). The mean mRNA levels of 5α-reductase in rats receiving kisspeptin+saline or kisspeptin+morphine did not decrease in comparison to morphine+saline (Figure 2). Kisspeptin+morphine did not increase the mean mRNA levels of 5α-reductase compared to kisspeptin+saline (Figure 2).

***Influence of morphine and kisspeptin on 5α-reductase mRNA levels of in the testis***

The mean mRNA levels of 5α-reductase in rats receiving morphine+saline, kisspeptin+saline or kisspeptin+morphine did not increase compared to saline+saline (Figure 2). The mean mRNA levels of 5α-reductase in rats receiving kisspeptin+saline or kisspeptin+morphine did not increase compared to morphine+saline (Figure 2). Kisspeptin+morphine did not increase the mean mRNA levels of 5α-reductase compared to kisspeptin+saline (Figure 2). 

## Discussion

Subcutaneous administration of morphine significantly increased hypothalamic aromatase mRNA levels. But it did not alter the aromatase gene expressions in the both testis and liver. The used dose of morphine in the present study was chosen based on our previous studies which showed inhibitory influence of 5 mg/Kg BW morphine on LH and testosterone hormones secretion in male Wistar rats (16). The present results derived of hypothalamic samples are consistent with Aloisi and her colleague’s study which demonstrated a significant increase of mRNA levels of *aromatase* in the diencephalon of Sprague- Dawley male adult rats following acute morphine injection (7). However, the results derived of testis and liver are partly inconsistent with the results of them (7). Their research indicated that morphine injection increased aromatase gene expression in testis and a non- significant increase of it in the liver following the acute injections of morphine in Sprague- Dawley male rats (7).

 Also, the present results showed that morphine significantly increased the hypothalamic 5α- reductase gene expression. But it did not changed 5α- reductase gene expression in testis and liver. The effects of morphine on hypothalamic 5α-reductase gene expression are according to Aloisi and her colleague’s study (7). However, the results derived of testis and liver are partly inconsistent with the results of them which reported a significant increase of 5α- reductase gene expression in liver and a non- significant increase of it in testis (7). Amini and her colleagues demonstrated that administration of morphine decreased testosterone levels in the serum, brain and spinal cord while morphine injection significantly increased dihydrotestosterone (DHT) and its metabolites (3 alpha-diol glucuronide) concentration in the serum of Spragoue- Dawley male adult rats (8). Also they showed that pretreatment with 5α- reductase inhibitor named finasteride significantly blocked the inhibitory effects of morphine on brain, spinal cord or serum concentration of testosterone and stimulatory effects of morphine on serum concentration of DHT (8). 

Hypothalamic estradiol or DHT derived from the action of aromatase and 5α- reductase enzymes, in addition to taking part in sexual differentiation of some areas of brain or setting up the negative feedback mechanism for regulation of basal plasma concentration of testosterone or neural protection are involved in the controlling metabolic functions. It has been completely established that both estradiol and androgen receptors are extensively expressed in liver of humans and rodents. Sex hormones exert metabolic effects in the liver and they are involved in the regulation of glucose and lipid homeostasis. Estrogens inhibit lipogenesis, gluconeogenesis and increasing lipolysis and glycogen storage. Androgens increase insulin receptors, glycogen synthesis and they decrease lipogenesis (3). In fact both estradiol and DHT produced in the liver prevent hepatic fat accumulation via factors mentioned. Also 5α- reductase is necessary and useful for clearance of steroid components including cortisol, corticosterone and glucocorticoids (17). In testis and prostate has been indicated that both aromatase and 5α- reductase enzymes are synthesized and the products of them have a role in puberty, spermatogenesis and growth of prostate and secondary sexual characteristics (18). So, in addition to hypothalamus, the effects of morphine were determined on aromatase or 5α- reductase genes expression in the liver and testis.

Unlike the hypothalamus, the effective dose of morphine did not exert any significant influences on aromatase or 5α- reductase genes expression in the liver and testis. To interpret the present results one could propose the synthesis levels of these genes might have different sensitivity to the dose of used drugs in several tissues. Also, it may be different tissues have a different sensitivity to the sexual hormones produced by these enzymes. Previous studies have shown that in the prostate and liver the expression of 5α- reductase gene is under control of positive feedback mechanism of testosterone and DHT (19, 20). While in the brain its synthesis is regulated by the negative feedback mechanism of testosterone and DHT (21). Also previous researches demonstrated that in the liver, in addition to metabolic effects, aromatase takes part in the metabolizing some drug (21). For example, aromatase plays a role in metabolizing and converting codeine to morphine in the liver (7). In intact rats, these enzymes synthesis, their sensitivity to drugs, steroid hormones and their functions are different (19- 21). So, different effects of 5mg/kg BW of on 5α- reductase and aromatase gene expression in liver and testis compared to brain might be due to different sensitivity of them to morphine used dose. 

Also, present results showed that kisspeptin did not significantly decrease the mean relative aromatase gene expressions in the hypothalamus, liver or testis. In the present research, the dose of kisspeptin was chosen based on previous study (12). There are not any previous reports to compare the present results of kisspeptin effects on *aromatase* gene expressions in any species. However, the previous studies are indicator of interaction of kisspeptin and aromatase signaling pathways. It has been established that kisspeptin and GPR54 receptor are expressed in liver and testes (22). Kisspeptin affect energy homeostasis, blood testosterone levels, puberty and spermatogenesis. Kisspeptin decrease energy expenditure and it is involved in the controlling insulin secretion, glucose and lipid metabolism and so on (22). A previous research demonstrated that kisspeptin neurons number in the brain of aromatase knock-out male rats is higher than wild type rats and their results suggested that aromatase might exerts an inhibitory effects on *Kiss1* gene expression (23). Also, our previous results showed that the effective dose of morphine used for inhibiting LH and testosterone secretion, significantly down- regulated the hypothalamic *Kiss1* mRNA levels while it did not alter *GPR54* (kisspeptin receptor) gene expression (12). The results of our previous study suggested that morphine might be indirectly involved in suppressing GnRH/LH and following testosterone synthesis via decreasing the activity of hypothalamic kisspeptin neurons (12). 

Kisspeptin signaling pathway have a role in the regulating and mediating the gonadal steroid hormones effects on both tonic (in males and females) and surge (in females) releases of GnRH/ LH hormones. Estradiol synthesized in the hypothalamus by the action of aromatase enzyme partly involved in the negative feedback mechanism control of HPG axis activity in males (22). In males including human, rodents and other animals indicated that injections of aromatase inhibitors disrupt the negative feedback mechanism control of GnRH/LH and testosterone while administration of estradiol to these animals or subjects return plasma testosterone levels into normal range (24). So, several studies highlighted the necessity of hypothalamic conversion of some testosterone to estradiol for correct regulation of basal blood levels of testosterone. Also estradiol receptor type α (ERα) gene expression has been shown on kisspeptin neurons (25). 

Also, the present results indicated that kisspeptin did not significantly decrease the mean relative 5α- reductase genes expression in the hypothalamus, liver or testis. There are not any previous reports to compare the present results of kisspeptin effects on 5α- reductase gene expressions in any species. However, the previous studies are indicator of interaction of kisspeptin and *5α- reductase* signaling pathways. It has been established that injection of DHT suppress the secretion of LH and testosterone in normal men and it decrease the hypothalamic GnRH gene expression and plasma LH concentration in rodents (26). Injections of DHT decrease the KiSS1 gene expressions in the ARC nucleus of hypothalamus of ovariectomized (OVX) rats. While it does not change KiSS1 gene expression of the AVPV nucleus in OVX or OVX- estradiol recieving rats (26). Subcutaneously implanting the DHT capsules in weaned female rats for 90 days significantly decreased the kisspeptin neurons number in both ARC and AVPV nucleus and androgen receptor highly expressed in kisspeptin neurons of ARC rather than AVPV nucleus (27). These results suggested that DHT disrupts the HPG axis partly via disorder of kisspeptin signaling pathway upstream GnRH neurons. 

Previous studies demonstrated a relation between aromatase 5α- reductase enzymes activity by focusing on the effects of DHT or aromatase on kisspeptin neural pathways (23- 27) and there is not any report about the influences of kisspeptin on 5α- reductase or aromatase activity or mRNA levels in any species. For the first time, we try to investigate the interaction of effective dose of morphine and kisspeptin stimulating the HPG axis on the aromatase or 5α- reductase genes expression levels in different peripheral tissues. The results of co- administration group showed that kisspeptin did not change the stimulatory effects of morphine on hypothalamic *aromatase* and *5α- reductase* genes expression. Also, injections of morphine plus kisspeptin did not alter mean relative aromatase or 5α- reductase genes expression in the testis and liver compared to control or morphine groups. To better understand the mechanisms of kisspeptin/GPR54 signaling system actions upstream aromatase or 5α- reductase neurons, and evaluating role of kisspeptin in controlling the opioid effects on aromatase or 5α- reductase, further studies by using kisspeptin or its receptor antagonist(peptide 234) doses higher than their effective doses used for stimulating HPG axis are needed to interpret precise effects of kisspeptin/GPR54 signaling pathway effects on aromatase or 5α- reductase enzymes synthesis or activity. 

## Conclusion

The inhibitory effective used dose of morphine on for stimulating LH and testosterone significantly up- regulates the hypothalamic mRNA levels of aromatase or 5α- reductase while it did not alter these enzymes genes expression levels in the liver and testis. The stimulatory effective used dose of kisspeptin on LH and testosterone did not change the aromatase or 5α- reductase genes expression levels in the hypothalamus, liver and testis. Also injection of kisspeptin plus morphine did not change effects of morphine on aromatase or 5α- reductase genes expression in the hypothalamus, liver and testis. These results suggest that different effects of morphine on aromatase or 5α- reductase genes expression levels in the liver and testis compared to brain might be due to the different sensitivity and functions of them to morphine used dose. 
